# Crystal structure of (*Z*)-3-allyl-5-(3-bromo­benzyl­idene)-2-sulfanyl­idene-1,3-thia­zolidin-4-one

**DOI:** 10.1107/S2056989015022884

**Published:** 2015-12-06

**Authors:** Rahhal El Ajlaoui, El Mostapha Rakib, Issam Forsal, Mohamed Saadi, Lahcen El Ammari

**Affiliations:** a Laboratoire de Chimie Organique et Analytique, Université Sultan Moulay Slimane, Faculté des Sciences et Techniques, Béni-Mellal, BP 523, Morocco; b Laboratoire de Chimie du Solide Appliquée, Faculté des Sciences, Université Mohammed V de Rabat, Avenue Ibn Battouta, BP. 1014, Rabat, Morocco

**Keywords:** crystal structure, rhodanine, hydrogen bonding

## Abstract

In the title compound, C_13_H_10_BrNOS_2_, the rhodanine (systematic name: 2-sulfanyl­idene-1,3-thia­zolidin-4-one) and the 3-bromo­benzyl­idene ring systems are inclined slightly, forming a dihedral angle of 5.86 (12)°. The rhodanine moiety is linked to an allyl group at the N atom and to the 3-bromo­benzyl­idene ring system. The allyl group, C=C—C, is nearly perpendicular to the mean plane through the rhodanine ring, maling a dihedral angle of 87.2 (5)°. In the crystal, mol­ecules are linked by pairs of C—H⋯O hydrogen bonds, forming inversion dimers with an *R*_2_^2^(10) ring motif.

## Related literature   

For pharmacological and biological activities of rhodanine-based mol­ecules, see: Tomasić & Masic (2009 ▸); Sortino *et al.* (2007 ▸); Kesel (2003 ▸); Capan *et al.* (1996 ▸); Momose *et al.* (1991 ▸); Kawakami *et al.* (1998 ▸); Insuasty *et al.* (2010 ▸). For the crystal structure of a related compound, see: El Ajlaoui *et al.* (2015 ▸).
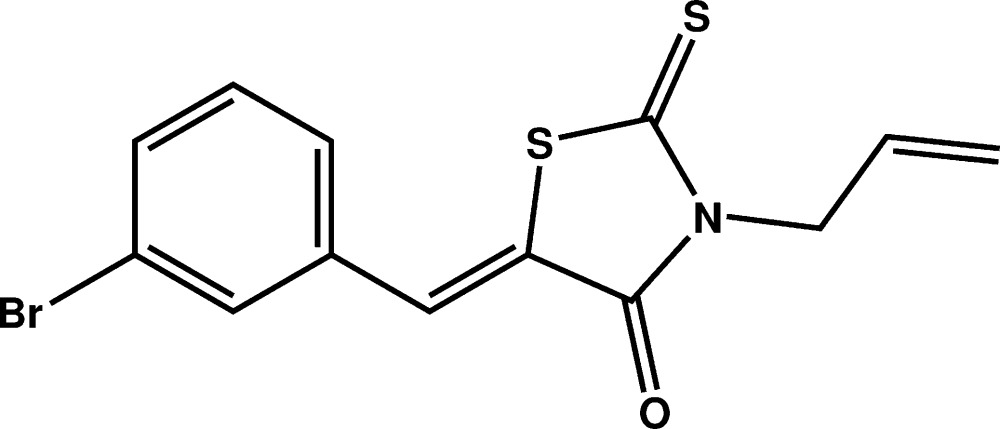


## Experimental   

### Crystal data  

C_13_H_10_BrNOS_2_
*M* *_r_* = 340.25
Triclinic, 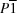

*a* = 5.4044 (6) Å
*b* = 11.2306 (13) Å
*c* = 11.7966 (13) Å
α = 80.100 (5)°
β = 84.912 (6)°
γ = 76.732 (6)°
*V* = 685.60 (13) Å^3^
*Z* = 2
Mo *K*α radiation
μ = 3.29 mm^−1^
*T* = 296 K
0.31 × 0.27 × 0.21 mm

### Data collection  

Bruker X8 APEX diffractometer
Absorption correction: multi-scan (*SADABS*; Bruker, 2009 ▸) *T*_min_ = 0.479, *T*_max_ = 0.746
25482 measured reflections
4181 independent reflections
2895 reflections with *I* > 2σ(*I*)
*R*_int_ = 0.044

### Refinement  

*R*[*F* ^2^ > 2σ(*F* ^2^)] = 0.039
*wR*(*F* ^2^) = 0.098
*S* = 1.01
4181 reflections
163 parameters
H-atom parameters constrained
Δρ_max_ = 0.95 e Å^−3^
Δρ_min_ = −0.71 e Å^−3^

### 

Data collection: *APEX2* (Bruker, 2009 ▸); cell refinement: *SAINT* (Bruker, 2009 ▸); data reduction: *SAINT*; program(s) used to solve structure: *SHELXS2014* (Sheldrick, 2008 ▸); program(s) used to refine structure: *SHELXL2014* (Sheldrick, 2015 ▸); molecular graphics: *ORTEPIII* (Burnett & Johnson, 1996 ▸), *ORTEP-3 for Windows* (Farrugia, 2012 ▸) and *PLATON* (Spek, 2009 ▸); software used to prepare material for publication: *publCIF* (Westrip, 2010 ▸).

## Supplementary Material

Crystal structure: contains datablock(s) I. DOI: 10.1107/S2056989015022884/su5249sup1.cif

Structure factors: contains datablock(s) I. DOI: 10.1107/S2056989015022884/su5249Isup2.hkl

Supporting information file. DOI: 10.1107/S2056989015022884/su5249Isup3.cml

**Fig. 1.**
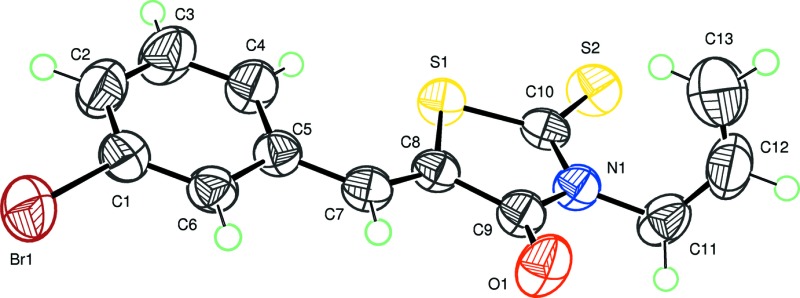
A view of the molecular structure of the title compound, with atom labelling. Displacement ellipsoids are drawn at the 50% probability level.

**Fig. 2.**
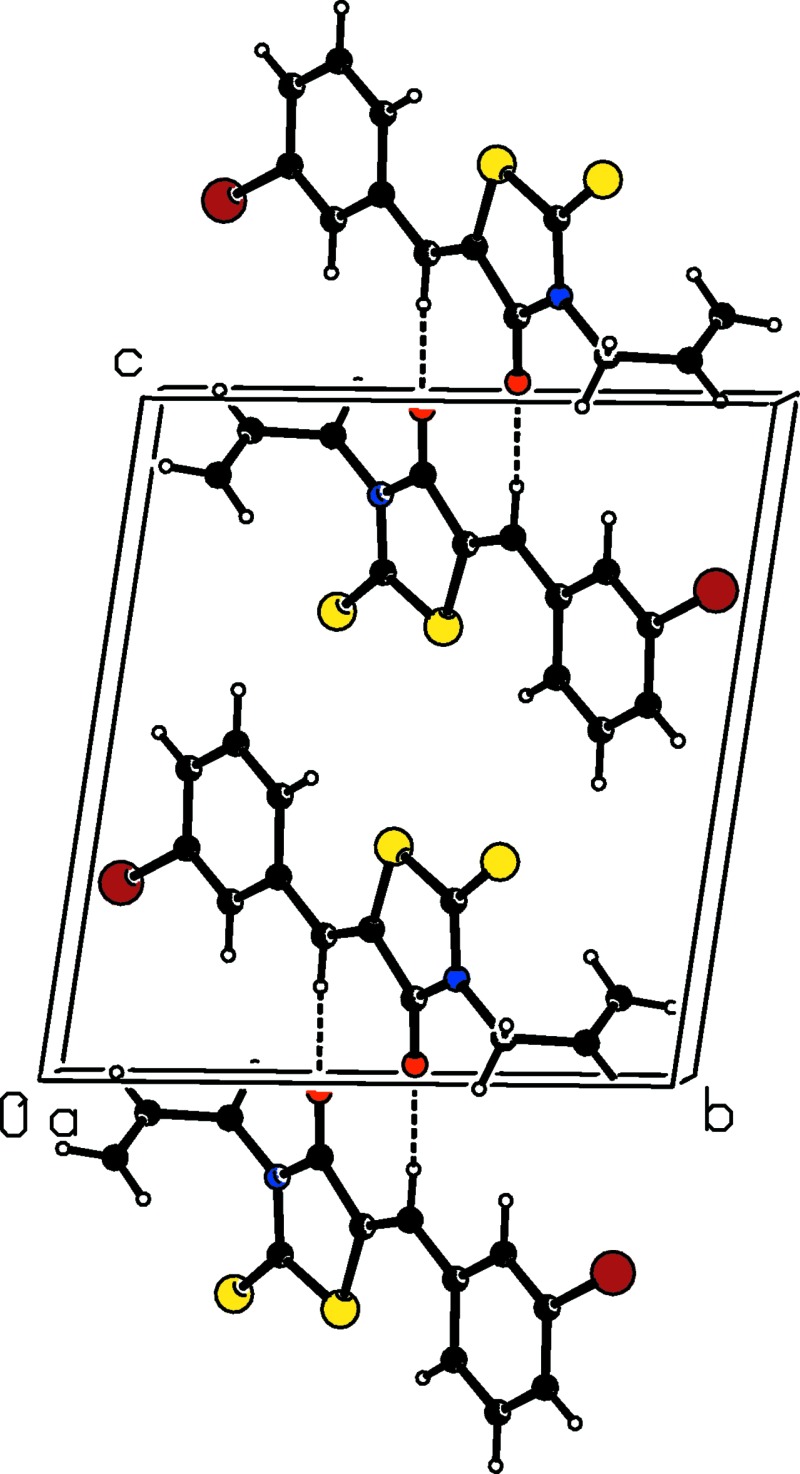
A view along the a axis of the crystal packing of the title compound, showing the hydrogen bonds as dashed lines (see Table 1).

. DOI: 10.1107/S2056989015022884/su5249fig1.tif
A view of the mol­ecular structure of the title compound, with atom labelling. Displacement ellipsoids are drawn at the 50% probability level.

a . DOI: 10.1107/S2056989015022884/su5249fig2.tif
A view along the *a* axis of the crystal packing of the title compound, showing the hydrogen bonds as dashed lines (see Table 1).

CCDC reference: 1439611

Additional supporting information:  crystallographic information; 3D view; checkCIF report

## Figures and Tables

**Table 1:** Hydrogen-bond geometry (Å, °)

*D*—H⋯*A*	*D*—H	H⋯*A*	*D*⋯*A*	*D*—H⋯*A*
C7—H7⋯O1^i^	0.93	2.42	3.310 (3)	159

Symmetry code: (i) 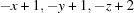.

## supplementary crystallographic information

### S1. Structural commentary

Rhodanine is an attractive scaffold unit because of its prestigious position in medicinal chemistry as it is responsible for numerous pharmacological and biological activities (Tomasic & Masic, 2009), *e.g.*, anti­microbial, anti­viral, anti­convulsant, anti­diabetic and anti­tumor activities (Sortino *et al.*, 2007; Kesel, 2003; Capan *et al.*, 1996; Momose *et al.*, 1991; Kawakami *et al.*, 1998; Insuasty *et al.* 2010). The unusual biological activity displayed by many rhodanine-based molecules have made them attractive synthetic targets.

The title compound, Fig. 1, is build up from a rhodanine ring (S1/N/1 C8–C10) linked to an allyl group (C11–C13) at the nitro­gen atom and to a 3-bromo­benzyl­idene ring system (C1—C6). The mean plane through the rhodanine ring is almost perpendicular to the allyl group (C11—C13) with a dihedral angle of 87.2 (5) °, and makes a dihedral angle of 5.86 (12)° with the 3-bromo­benzyl­idene ring. A very similar arrangement has been observed in the crystal structure of (*Z*)-3-allyl-5-(4-methyl-benzyl­idene)-2-thioxo­thia­zolidin-4-one, but with disorder in the allyl group (El Ajlaoui *et al.*, 2015).

In the crystal, molecules are linked by a pair of C—H···O hydrogen bonds forming inversion dimers with an *R*^2^_2_(10) ring motif (Table 1 and Fig. 2).

### S2. Synthesis and crystallization

To a solution of 3-allyl­rhodanine (1.15 mmol, 0.2 g) in 10 ml of THF, (3-bromo­benzyl­idene)-4-methyl-5-oxopyrazolidin-2-ium-1-ide (1.38 mmol) was added and the mixture refluxed for 8 h, monitored by TLC. On completion of the reaction, with a yellow spot (TLC Rf = 0.3, using hexane/ethyl acetate 1:9) generated cleanly, the solvent was evaporated *in vacuo*. The crude product was purified on silica gel using hexane:ethyl acetate (1:9) as eluent. The title compound was recrystallized from ethanol giving colourless block-like crystals (yield: 76%; m.p. 390 K).

### S3. Refinement

Crystal data, data collection and structure refinement details are summarized in Table 2. H atoms were located in a difference Fourier map and treated as riding: C–H = 0.93–0.97 Å with *U*_iso_(H) = 1.2*U*_eq_(C). Two reflections, (0 1 0) and (0 0 1), affected by the beam-stop were removed during the final cycles of refinement.

### Crystal data

**Table:**

C_13_H_10_BrNOS_2_	*F*(000) = 340
*M**_r_* = 340.25	*D*_x_ = 1.648 Mg m^−^^3^
Triclinic, *P*1	Melting point: 390 K
*a* = 5.4044 (6) Å	Mo *K*α radiation, λ = 0.71073 Å
*b* = 11.2306 (13) Å	Cell parameters from 4181 reflections
*c* = 11.7966 (13) Å	θ = 2.8–30.5°
α = 80.100 (5)°	µ = 3.29 mm^−^^1^
β = 84.912 (6)°	*T* = 296 K
γ = 76.732 (6)°	Block, colourless
*V* = 685.60 (13) Å^3^	0.31 × 0.27 × 0.21 mm
*Z* = 2	

### Data collection

**Table:**

Bruker X8 APEX diffractometer	4181 independent reflections
Radiation source: fine-focus sealed tube	2895 reflections with *I* > 2σ(*I*)
Graphite monochromator	*R*_int_ = 0.044
φ and ω scans	θ_max_ = 30.5°, θ_min_ = 2.8°
Absorption correction: multi-scan (*SADABS*; Bruker, 2009)	*h* = −7→7
*T*_min_ = 0.479, *T*_max_ = 0.746	*k* = −16→16
25482 measured reflections	*l* = −16→16

### Refinement

**Table:**

Refinement on *F*^2^	0 restraints
Least-squares matrix: full	Hydrogen site location: inferred from neighbouring sites
*R*[*F*^2^ > 2σ(*F*^2^)] = 0.039	H-atom parameters constrained
*wR*(*F*^2^) = 0.098	*w* = 1/[σ^2^(*F*_o_^2^) + (0.0356*P*)^2^ + 0.492*P*] where *P* = (*F*_o_^2^ + 2*F*_c_^2^)/3
*S* = 1.01	(Δ/σ)_max_ = 0.001
4181 reflections	Δρ_max_ = 0.95 e Å^−^^3^
163 parameters	Δρ_min_ = −0.71 e Å^−^^3^

### Special details

**Table:**

Geometry. All e.s.d.'s (except the e.s.d. in the dihedral angle between two l.s. planes) are estimated using the full covariance matrix. The cell e.s.d.'s are taken into account individually in the estimation of e.s.d.'s in distances, angles and torsion angles; correlations between e.s.d.'s in cell parameters are only used when they are defined by crystal symmetry. An approximate (isotropic) treatment of cell e.s.d.'s is used for estimating e.s.d.'s involving l.s. planes.

### Fractional atomic coordinates and isotropic or equivalent isotropic displacement parameters (Å^2^)

**Table:**

	*x*	*y*	*z*	*U*_iso_*/*U*_eq_	
C1	0.7585 (5)	0.8474 (2)	0.6704 (2)	0.0449 (5)	
C2	0.7277 (6)	0.8621 (3)	0.5535 (2)	0.0578 (7)	
H2	0.7974	0.9201	0.5025	0.069*	
C3	0.5914 (6)	0.7891 (3)	0.5138 (2)	0.0625 (8)	
H3	0.5704	0.7977	0.4352	0.075*	
C4	0.4861 (5)	0.7039 (3)	0.5885 (2)	0.0516 (6)	
H4	0.3934	0.6562	0.5601	0.062*	
C5	0.5173 (4)	0.6882 (2)	0.70721 (19)	0.0379 (5)	
C6	0.6565 (4)	0.7623 (2)	0.7469 (2)	0.0392 (5)	
H6	0.6799	0.7540	0.8253	0.047*	
C7	0.4177 (4)	0.5987 (2)	0.79133 (19)	0.0386 (5)	
H7	0.4638	0.5940	0.8663	0.046*	
C8	0.2693 (4)	0.5212 (2)	0.77866 (18)	0.0358 (4)	
C9	0.1983 (4)	0.4349 (2)	0.87829 (19)	0.0402 (5)	
C10	−0.0140 (4)	0.3884 (2)	0.7315 (2)	0.0385 (5)	
C11	−0.0559 (5)	0.2749 (3)	0.9303 (2)	0.0538 (7)	
H11A	−0.2218	0.2691	0.9094	0.065*	
H11B	−0.0769	0.3018	1.0051	0.065*	
C12	0.1173 (8)	0.1500 (3)	0.9393 (3)	0.0717 (9)	
H12	0.0739	0.0898	0.9972	0.086*	
C13	0.3193 (8)	0.1155 (3)	0.8769 (3)	0.0845 (11)	
H13A	0.3724	0.1716	0.8176	0.101*	
H13B	0.4125	0.0343	0.8909	0.101*	
N1	0.0372 (4)	0.36716 (18)	0.84606 (16)	0.0398 (4)	
O1	0.2673 (4)	0.42021 (19)	0.97577 (14)	0.0579 (5)	
S1	0.13666 (12)	0.50102 (6)	0.65523 (5)	0.04169 (14)	
S2	−0.18899 (14)	0.31940 (7)	0.67061 (6)	0.05491 (18)	
Br1	0.94248 (6)	0.94874 (3)	0.72543 (3)	0.06670 (13)	

### Atomic displacement parameters (Å^2^)

**Table:**

	*U*^11^	*U*^22^	*U*^33^	*U*^12^	*U*^13^	*U*^23^
C1	0.0478 (14)	0.0440 (13)	0.0461 (13)	−0.0140 (11)	−0.0009 (10)	−0.0110 (10)
C2	0.0697 (18)	0.0600 (17)	0.0465 (14)	−0.0269 (14)	0.0026 (13)	−0.0013 (12)
C3	0.080 (2)	0.079 (2)	0.0341 (13)	−0.0327 (17)	−0.0068 (13)	−0.0022 (13)
C4	0.0625 (16)	0.0627 (16)	0.0376 (12)	−0.0268 (13)	−0.0082 (11)	−0.0086 (11)
C5	0.0402 (12)	0.0407 (12)	0.0345 (11)	−0.0097 (9)	−0.0058 (9)	−0.0077 (9)
C6	0.0424 (12)	0.0420 (12)	0.0356 (11)	−0.0106 (10)	−0.0030 (9)	−0.0105 (9)
C7	0.0418 (12)	0.0450 (12)	0.0319 (10)	−0.0102 (10)	−0.0087 (9)	−0.0097 (9)
C8	0.0381 (11)	0.0397 (11)	0.0315 (10)	−0.0072 (9)	−0.0077 (8)	−0.0093 (9)
C9	0.0434 (12)	0.0455 (12)	0.0359 (11)	−0.0142 (10)	−0.0071 (9)	−0.0091 (9)
C10	0.0346 (11)	0.0436 (12)	0.0403 (12)	−0.0059 (9)	−0.0075 (9)	−0.0152 (9)
C11	0.0588 (16)	0.0713 (18)	0.0417 (13)	−0.0359 (14)	0.0026 (11)	−0.0107 (12)
C12	0.106 (3)	0.0573 (18)	0.0569 (18)	−0.0387 (18)	−0.0014 (18)	0.0050 (14)
C13	0.098 (3)	0.060 (2)	0.082 (3)	−0.0004 (19)	−0.008 (2)	0.0036 (18)
N1	0.0432 (10)	0.0464 (11)	0.0345 (9)	−0.0159 (9)	−0.0060 (8)	−0.0085 (8)
O1	0.0756 (13)	0.0754 (13)	0.0335 (9)	−0.0392 (11)	−0.0177 (8)	0.0004 (8)
S1	0.0471 (3)	0.0495 (3)	0.0328 (3)	−0.0148 (3)	−0.0131 (2)	−0.0066 (2)
S2	0.0547 (4)	0.0703 (4)	0.0517 (4)	−0.0268 (3)	−0.0135 (3)	−0.0194 (3)
Br1	0.0796 (2)	0.0688 (2)	0.0663 (2)	−0.04359 (17)	0.00513 (15)	−0.01814 (15)

### Geometric parameters (Å, º)

**Table:**

C1—C6	1.374 (3)	C8—S1	1.749 (2)
C1—C2	1.381 (4)	C9—O1	1.213 (3)
C1—Br1	1.896 (2)	C9—N1	1.394 (3)
C2—C3	1.380 (4)	C10—N1	1.372 (3)
C2—H2	0.9300	C10—S2	1.631 (2)
C3—C4	1.374 (4)	C10—S1	1.739 (2)
C3—H3	0.9300	C11—N1	1.453 (3)
C4—C5	1.401 (3)	C11—C12	1.489 (5)
C4—H4	0.9300	C11—H11A	0.9700
C5—C6	1.401 (3)	C11—H11B	0.9700
C5—C7	1.447 (3)	C12—C13	1.283 (5)
C6—H6	0.9300	C12—H12	0.9300
C7—C8	1.345 (3)	C13—H13A	0.9300
C7—H7	0.9300	C13—H13B	0.9300
C8—C9	1.472 (3)		
			
C6—C1—C2	121.4 (2)	C9—C8—S1	109.66 (16)
C6—C1—Br1	119.79 (18)	O1—C9—N1	122.5 (2)
C2—C1—Br1	118.8 (2)	O1—C9—C8	127.0 (2)
C3—C2—C1	118.7 (2)	N1—C9—C8	110.44 (18)
C3—C2—H2	120.7	N1—C10—S2	126.32 (19)
C1—C2—H2	120.7	N1—C10—S1	110.91 (16)
C4—C3—C2	121.1 (3)	S2—C10—S1	122.77 (14)
C4—C3—H3	119.5	N1—C11—C12	113.0 (2)
C2—C3—H3	119.5	N1—C11—H11A	109.0
C3—C4—C5	120.6 (2)	C12—C11—H11A	109.0
C3—C4—H4	119.7	N1—C11—H11B	109.0
C5—C4—H4	119.7	C12—C11—H11B	109.0
C6—C5—C4	118.1 (2)	H11A—C11—H11B	107.8
C6—C5—C7	117.89 (19)	C13—C12—C11	127.9 (3)
C4—C5—C7	124.0 (2)	C13—C12—H12	116.1
C1—C6—C5	120.2 (2)	C11—C12—H12	116.1
C1—C6—H6	119.9	C12—C13—H13A	120.0
C5—C6—H6	119.9	C12—C13—H13B	120.0
C8—C7—C5	130.5 (2)	H13A—C13—H13B	120.0
C8—C7—H7	114.8	C10—N1—C9	116.30 (19)
C5—C7—H7	114.8	C10—N1—C11	123.3 (2)
C7—C8—C9	120.37 (19)	C9—N1—C11	120.27 (19)
C7—C8—S1	129.97 (18)	C10—S1—C8	92.61 (11)

### Hydrogen-bond geometry (Å, º)

**Table:**

*D*—H···*A*	*D*—H	H···*A*	*D*···*A*	*D*—H···*A*
C7—H7···O1^i^	0.93	2.42	3.310 (3)	159

Symmetry code: (i) −*x*+1, −*y*+1, −*z*+2.
