# Supplementary figures and images for: Crystal structure of (Z)-3-allyl-5-(3-bromo­benzyl­idene)-2-sulfanyl­idene-1,3-thia­zolidin-4-one
Source: Acta Crystallogr E Crystallogr Commun. 2015 Dec 6;71(Pt 12):o1010–1. doi: 10.1107/S2056989015022884 (PMC4719950; doi:10.1107/S2056989015022884)

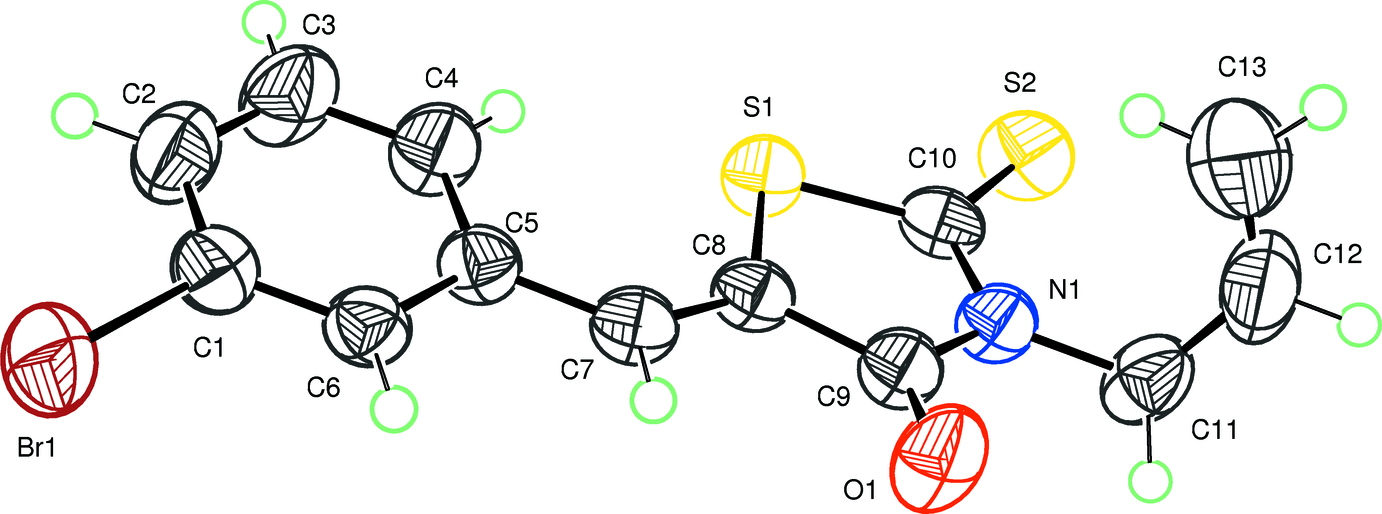

Supplement: Supplementary file 4 [file e-71-o1010-fig1.tif]

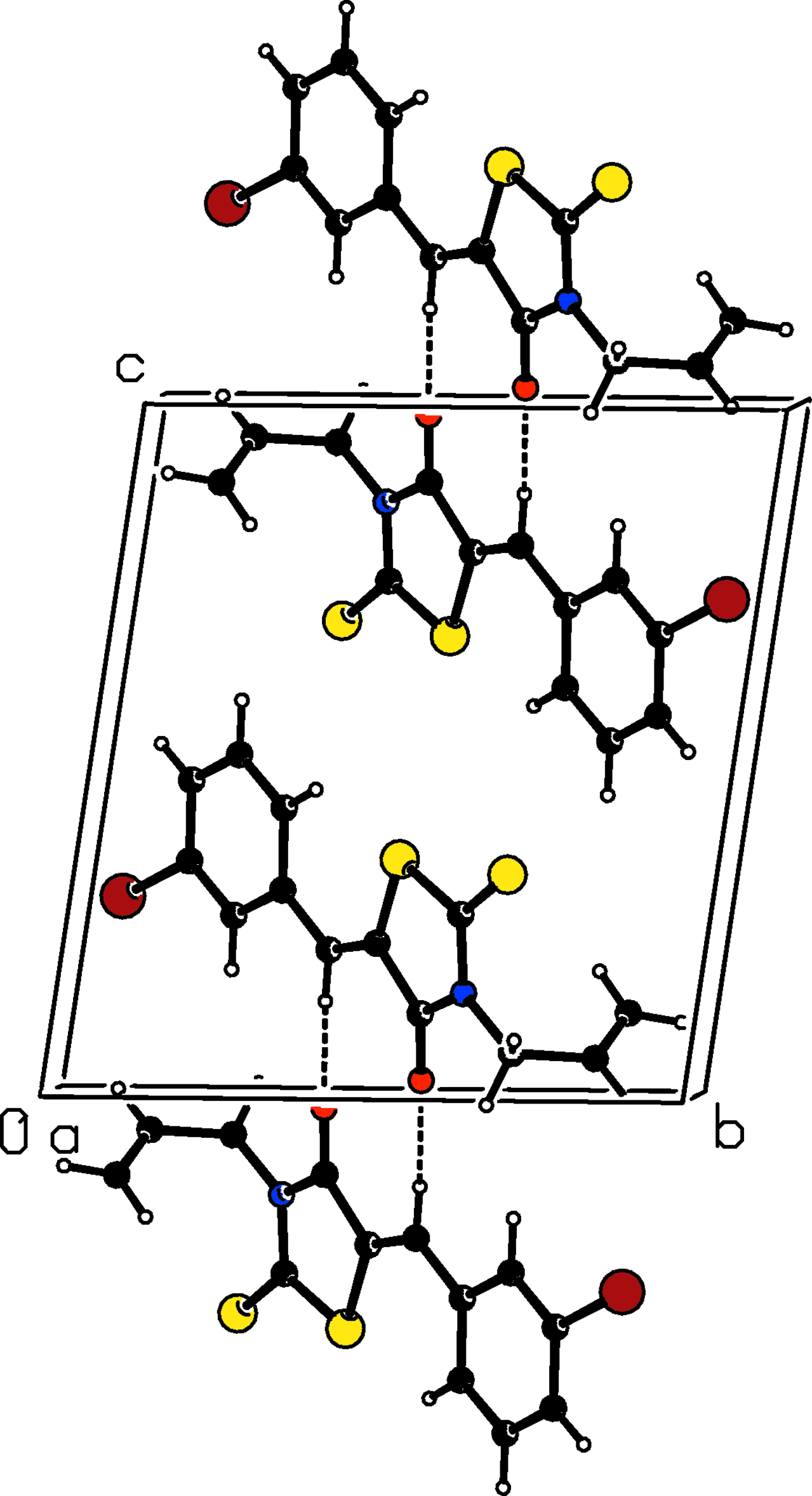

Supplement: Supplementary file 5 [file e-71-o1010-fig2.tif]
